# Synthesis and Structure-Activity Relationships of Novel Ecdysteroid Dioxolanes as MDR Modulators in Cancer

**DOI:** 10.3390/molecules181215255

**Published:** 2013-12-10

**Authors:** Ana Martins, József Csábi, Attila Balázs, Diána Kitka, Leonard Amaral, József Molnár, András Simon, Gábor Tóth, Attila Hunyadi

**Affiliations:** 1 Department of Medical Microbiology and Immunobiology, University of Szeged, Dóm tér 9, Szeged 6720, Hungary; E-Mails: kitka.diana@gmail.com (D.K.); molnar.jozsef@med.u-szeged.hu (J.M.); 2 Unidade de Parasitologia e Microbiologia Médica, Institute of Hygiene and Tropical Medicine, Universidade Nova de Lisboa, Rua da Junqueira 100, Lisbon 1349-008, Portugal; 3 Institute of Pharmacognosy, Faculty of Pharmacy, University of Szeged, Eötvös u. 6, Szeged 6720, Hungary; E-Mail: csjoco88@gmail.com; 4 Ubichem Research Ltd., Illatos út 33, Budapest H-1097, Hungary; E-Mail: abalazs@ubichempharma.com; 5 Center for Malaria and Other Tropical Diseases (CMDT), Institute of Hygiene and Tropical Medicine, Universidade Nova de Lisboa, Rua da Junqueira 100, Lisbon 1349-008, Portugal; E-Mail: LAmaral@ihmt.unl.pt; 6 Department of Inorganic and Analytical Chemistry, Budapest University of Technology and Economics, Szt. Gellért tér 4, Budapest H-1111, Hungary; E-Mails: andras.simon@mail.bme.hu (A.S.); drtothgabor@t-online.hu (G.T.)

**Keywords:** ecdysteroids, 20-hydroxyecdysone, acetonide, dioxolane, stereochemistry, cancer, multi-drug resistance, P-glycoprotein, ABCB1 transporter, efflux pump

## Abstract

Ecdysteroids, molting hormones of insects, can exert several mild, non-hormonal bioactivities in mammals, including humans. In a previous study, we have found a significant effect of certain derivatives on the ABCB1 transporter mediated multi-drug resistance of a transfected murine leukemia cell line. In this paper, we present a structure-activity relationship study focused on the apolar dioxolane derivatives of 20-hydroxyecdysone. Semi-synthesis and bioactivity of a total of 32 ecdysteroids, including 20 new compounds, is presented, supplemented with their complete ^1^H- and ^13^C-NMR signal assignment.

## 1. Introduction

Ecdysteroids represent a large family of steroid hormones that play a crucial role in arthropods’ physiology. The most abundant representative of these compounds, 20-hydroxyecdysone (20E), regulates the reproduction, embryogenesis, diapause and molting of arthropods [1]. Their role in plants is still to be fully understood, but it had been suggested that they have high importance in several plants as defensive agents against non-adapted herbivores [2]. An estimated 5%–6% of the terrestrial plant species accumulate detectable levels of ecdysteroids, among which *Ajuga*, *Serratula* and *Silene* spp., containing high amounts of these compounds, are good sources of ecdysteroids of herbal origin [3].

Ecdysteroids generally retain the cholesterol-originated side-chain, typically contain 27–29 carbon atoms and are substituted with 4–8 hydroxyl groups. Their A/B ring junction is usually *cis*, and a characteristic 7-en-6-one (α,β-unsaturated ketone) chromophore group is present in their B-ring [4]. Due to their significantly different structure as compared to the vertebrate steroid hormones, these compounds have no hormonal effects in humans [5]. On the other hand, a number of beneficial metabolic effects are attributed to them [4,5,6], which has encouraged the production and worldwide marketing of many food supplements, mainly containing the isolated ecdysteroid compound 20E [6].

In our recent studies, we found that certain ecdysteroid derivatives significantly decrease the resistance of a multi-drug resistant (MDR) murine leukemia cell line expressing the human ABCB1 transporter to doxorubicin, a known chemotherapeutic agent that is a substrate of the ABCB1 transporter, and we discussed the possible mechanisms that might be involved in this activity [7]. Based on the observed structure-activity relationships of the isolated and semi-synthesized ecdysteroids, 20-hydroxyecdysone 2,3;20,22-diacetonide (**1**) was chosen as the most promising lead. Although the acetonide moiety is generally utilized as an easy-to-remove protecting group for vicinal diols (which removal needs a strong acidic environment), it is also an important structural element of certain drugs, such as for example triamcinolone acetonide, which is not a pro-drug for triamcinolone but has distinctly different pharmacological and pharmacokinetical properties [8]. Following our previous work, we have synthesized several additional dioxolane derivatives and thoroughly discussed their structure elucidation and stereochemistry [9]. In the study reported herein we present the synthesis, structure elucidation and MDR-modulating activity of 32 ecdysteroid dioxolanes, including 20 new derivatives, and provide further insights on the structure-activity relationships of these compounds. 

## 2. Results and Discussion

### 2.1. Semi-Synthesis

**Figure 1:**
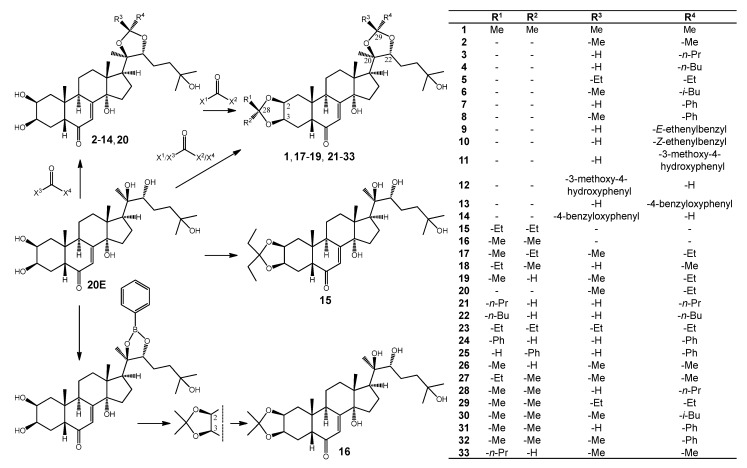
Semi-synthetic transformations of 20E and structures of the products obtained. Substituents of the reagent oxo-compound (X^1^/X^2^ and X^3^/X^4^) typically correspond to R^1^/R^2^ and R^2^/R^3^, respectively, except for compounds **18** and **19**, where the reagent was methyl-ethyl ketone. **15** was obtained as a side product from the synthesis of **23**.

Based on the formation of a common protecting group of vicinal diols, the acetonide, 32 compounds containing one or two dioxolane rings were synthesized from 20-hydroxyecysone with various aldehydes and ketones in the presence of phosphomolybdic acid. A summary of the reactions performed and the structures of the products obtained are presented in Figure 1. ^1^H- and ^13^C-NMR data of the new compounds are presented in Table 1, Table 2 and Table 3. To facilitate the comparison between the NMR signals of structurally analogous hydrogen and carbon atoms in the different dioxolane compounds, we applied a special numbering system for the central atoms (C-28 and C-29) of the 2,3- and 20,22-dioxolane structures. Compounds containing similar number of carbon atoms are presented in one table, and compounds with the highest structural similarity are presented in neighbouring columns.

**Table 1:** ^1^H- and ^13^C-NMR shifts of compounds **4**, **6**, **22**, **30**, **33**, **9** and **10**; in ppm, in methanol-*d*_4_.

Atom no.	4		6		22		30		33		9		10	
	H	C	H	C	H	C	H	C	H	C	H	C	H	C
1α β	1.43 1.79	37.5	1.43 1.79	37.5	1.17 2.00	39.6	1.22 1.98	38.8	1.16 1.99	39.6	1.43 1.79	37.5	1.44 1.79	37.5
2	3.84	68.8	3.84	68.8	4.21	72.8	4.26	73.6	4.21	72.8	3.84	68.8	3.84	68.8
3	3.95	68.6	3.95	68.6	4.11	75.0	4.30	73.2	4.12	75.1	3.95	68.6	3.95	68.6
4α β	1.74 1.70	32.9	1.75 1.70	32.9	2.01 1.97	27.8	1.97 1.97	27.8	2.00 2.00	27.8	1.74 1.71	32.9	1.74 1.71	33.0
5	2.39	51.8	2.39	51.8	2.24	52.6	2.24	52.6	2.24	52.6	2.39	51.9	2.39	51.9
6	-	206.4	-	206.5	-	205.4	-	205.7	-	205.5	-	206.4	-	206.5
7	5.81	122.2	5.82	122.2	5.80	121.9	5.80	121.9	5.80	121.9	5.82	122.3	5.82	122.3
8	-	167.6	-	167.7	-	167.0	-	167.0	-	167.1	-	167.6	-	167.6
9	3.15	35.2	3.15	35.2	2.94	36.3	2.93	35.8	2.94	36.3	3.16	35.3	3.16	35.3
10	-	39.0	-	39.3	-	38.8	-	38.9	-	38.8	-	39.3	-	39.4
11α β	1.69 1.80	21.6	1.69 1.80	21.6	1.69 1.78	21.6	1.66 1.77	21.7	1.70 1.79	21.8	1.68 1.80	21.6	1.71 1.81	21.6
12α β	1.84 2.11	32.3	1.86 2.12	32.4	1.84 2.10	32.3	1.85 2.11	32.4	1.83 2.11	32.4	1.86 2.13	32.3	1.87 2.13	32.3
13	-	48.4	-	48.6	-	48.7	-	48.8	-	48.8	-	48.4	-	48.5
14	-	85.3	-	85.4	-	85.3	-	85.3	-	85.4	-	85.3	-	85.3
15α β	1.95 1.62	31.9	1.95 1.62	31.8	1.96 1.61	31.7	1.95 1.61	31.7	1.95 1.59	31.7	1.97 1.63	31.9	2.01 1.64	31.9
16α β	1.94 1.89	22.7	2.03 1.89	22.5	1.94 1.90	22.7	2.03 1.86	22.5	2.03 1.86	22.5	2.00 1.94	22.8	1.93 1.90	22.9
17	2.35	51.4	2.32	50.6	2.36	51.4	2.31	50.6	2.31	50.6	2.40	51.4	2.40	51.4
18	0.86	17.8	0.83	17.9	0.86	17.8	0.82	17.9	0.82	17.8	0.89	17.8	0.92	17.8
19	0.97	24.6	0.96	24.6	0.97	24.2	0.96	24.1	0.96	24.1	0.96	24.6	0.97	24.6
20	-	85.5	-	85.7	-	84.9	-	85.7	-	85.9	-	85.8	-	85.9
21	1.14	23.7	1.17	23.0	1.14	23.7	1.17	23.0	1.18	22.7	1.24	23.5	1.24	23.5
22	3.63	85.0	3.65	82.4	3.64	85.5	3.65	82.4	3.69	83.4	3.73	85.7	3.69	85.8
23	1.57	24.7	1.73	24.8	1.55	24.8	1.50	24.8	1.53	24.8	1.62	24.8	1.63	24.8
24	1.76 1.50	42.3	1.73 1.50	42.3	1.77 1.51	42.3	1.73 1.48	42.3	1.73 1.50	42.3	1.79 1.53	42.2	1.79 1.53	42.3
25	-	71.2	-	71.2	-	71.2	-	71.2	-	71.2	-	71.2	-	71.2
26	1.20	29.1	1.20	29.1	1.20	29.1	1.19	29.1	1.20	29.1	1.21	29.1	1.21	29.1
27	1.21	29.6	1.21	29.6	1.21	29.6	1.21	29.6	1.21	29.6	1.22	29.7	1.22	29.6
28	-	-	-	-	-	105.9	-	109.6	-	105.7	-	-	-	-
29	-	105.6	-	109.8	-	105.6	-	109.8	-	108.1	-	105.2	-	100.6
R^1^ 1 2 3 4	-	-	-	-	1.67 1.41 1.39 0.93	36.3 27.5 23.8 14.5	1.47 -	28.9 -	1.63 1.46 0.97	38.8 18.8 14.5	-	-	-	-
R^2^ 1	-	-	-	-	4.93	-	1.32	26.8	4.94	-	-	-	-	-
R^3^ 1	4.91	-	1.29	25.1	4.91	-	1.29	25.1	1.32	27.3	5.41	-	5.47	-
R^4^ 1 2 3 4	1.59 1.38 1.38 0.92	36.4 27.5 23.8 14.5	1.64 1.45 1.80 0.94 0.97	52.3 26.1 25.0 24.0	1.59 1.38 1.38 0.92	36.4 27.6 23.8 14.5	1.65 1.44 1.80 0.94 0.97	52.2 26.2 25.0 24.0	1.39	29.5	6.13 6.74	128.5 135.7	5.67 6.78	130.9 135.8

**Table 2:** ^1^H- and ^13^C-NMR shifts of compounds **16**–**20** and **26**–**27**; in ppm, in methanol-*d*_4_.

Atom no.	16		26		27		17		18		19		20	
	H	C	H	C	H	C	H	C	H	C	H	C	H	C
1α β	1.22 1.99	38.9	1.16 2.02	39.8	1.22 1.98	39.0	1.25 1.99	39.0	1.22 1.98	39.0	1.17 2.02	39.8	1.43 1.79	37.5
2	4.26	73.6	4.21	73.1	4.27	73.2	4.25	73.6	4.27	73.2	4.21	73.1	3.84	68.8
3	4.31	73.3	4.13	75.3	4.33	72.8	4.27	73.3	4.33	73.8	4.13	75.3	3.95	68.6
4α β	1.97 1.97	27.8	2.00 2.00	27.9	1.98 1.98	27.8	1.97 1.97	27.8	1.98 1.98	27.8	2.00 2.00	27.8	1.76 1.72	33.0
5	2.24	52.6	2.25	52.7	2.23	52.6	2.25	52.5	2.22	52.7	2.25	52.6	2.39	51.9
6	-	205.7	-	205.8	-	205.7	-	205.6	-	205.7	-	205.5	-	206.5
7	5.79	121.9	5.80	121.9	5.79	121.9	5.79	121.9	5.79	121.9	5.80	121.9	5.82	122.2
8	-	167.3	-	167.1	-	167.0	-	166.9	-	167.0	-	167.0	-	167.7
9	2.94	35.9	2.94	36.2	2.93	36.1	2.93	35.9	2.93	36.1	2.94	36.2	3.15	35.2
10	-	39.8	-	38.9	-	38.9	-	38.9	-	38.9	-	38.9	-	39.3
11α β	1.67 1.78	21.7	1.67 1.77	21.7	1.68 1.78	21.8	1.68 1.77	21.7	1.68 1.78	21.7	1.66 1.78	21.8	1.70 1.81	21.6
12α β	1.87 2.12	32.6	1.85 2.10	32.4	1.84 2.10	32.4	1.85 2.11	32.5	1.83 2.10	32.3	1.85 2.11	32.5	1.86 2.12	32.5
13	-	49.0	-	49.3	-	48.8	-	48.8	-	48.7	-	48.8	-	48.7
14	-	85.3	-	85.3	-	85.9	-	85.3	-	85.4	-	85.3	-	85.4
15α β	1.97 1.59	31.8	1.95 1.61	31.7	1.96 1.61	31.7	1.96 1.61	31.7	1.95 1.61	31.7	1.97 1.62	31.7	1.97 1.61	31.8
16α β	1.98 1.72	21.6	2.03 1.87	22.5	2.03 1.86	22.7	2.04 1.88	22.6	1.94 1.88	22.7	2.04 1.87	22.6	2.03 1.88	22.6
17	2.39	50.7	2.31	50.6	2.31	50.6	2.32	50.7	2.35	51.4	2.32	50.7	2.32	50.7
18	0.88	18.1	0.82	17.8	0.82	17.8	0.83	17.8	0.85	17.7	0.83	17.8	0.83	17.8
19	0.96	24.1	0.96	24.1	0.96	24.2	0.97	24.2	0.96	24.1	0.96	24.1	0.96	24.6
20	-	78.0	-	85.3	-	85.3	-	85.5	-	85.5	-	85.6	-	85.6
21	1.19	21.2	1.18	22.7	1.18	22.7	1.16	231	1.15	23.8	1.16	23.0	1.16	23.0
22	3.33	78.5	3.68	83.4	3.69	83.4	3.71	83.1	3.65	83.8	3.71	83.1	3.71	83.1
23	1.66 1.27	27.5	1.52	24.8	1.53	24.8	1.52	24.5	1.56	24.8	1.52	24.8	1.53	24.8
24	1.78 1.44	42.5	1.73 1.49	42.3	1.73 1.48	42.3	1.72 1.49	42.3	1.74 1.50	42.3	1.73 1.50	42.3	1.74 1.50	42.3
25	-	71.4	-	71.2	-	71.2	-	71.2	-	71.2	-	71.2	-	71.2
26	1.19	29.1	1.20	29.1	1.20	29.1	1.20	29.2	1.20	29.0	1.20	29.1	1.20	29.1
27	1.20	29.8	1.21	29.6	1.21	29.6	1.21	29.6	1.21	29.6	1.21	29.6	1.21	29.5
28	-	109.6	-	102.7	-	111.5	-	111.7	-	111.5	-	102.8	-	-
29	-	-	-	108.0	-	108.1	-	110.0	-	102.5	-	110.0	-	110.0
R^1^ 1 2	1.47	28.9	1.38	21.9	1.75 0.99	35.7 9.4	1.41	25.8	1.73 0.99	35.7 9.4	1.38	21.9	-	-
R^2^ 1 2	1.32	26.7	5.09	-	1.27	23.6	1.61 0.91	39.5 9.1	1.27	23.6	5.10	-	-	-
R^3^ 1	-	-	1.32	27.3	1.32	27.6	1.27	24.2	5.06	-	1.27	24.2	1.27	24.2
R^4^ 1 2	-	-	1.39	29.4	1.39	29.5	1.63 0.96	36.2 9.6	1.30	22.1	1.64 0.95	36.2 9.6	1.65 0.95	36.2 9.6

**Table 3:** ^1^H- and ^13^C-NMR shifts of compounds **11**–**14**; **31** and **32**; in ppm, in methanol-*d*_4_.

Atom no.	11		12		13		14		31		32	
	H	C	H	C	H	C	H	C	H	C	H	C
1α β	1.43 1.80	37.5	1.43 1.80	37.5	1.43 1.79	37.5	1.43 1.80	37.5	1.22 1.97	38.8	1.23 1.99	38.8
2	3.84	68.8	3.84	68.8	3.84	68.8	3.84	68.8	4.27	73.6	4.26	73.6
3	3.95	68.2	3.95	68.6	3.95	68.6	3.95	68.6	4.30	73.2	4.30	73.3
4α β	1.72 1.72	33.0	1.72 1.72	33.0	1.72 1.72	32.9	1.72 1.72	33.0	1.97 1.97	27.8	1.98 1.98	27.8
5	2.38	51.9	2.38	51.9	2.39	51.8	2.38	51.9	2.24	52.5	2.26	52.5
6	-	206.5	-	206.5	-	206.5	-	206.5	-	205.5	-	205.7
7	5.82	122.3	5.81	122.3	5.82	122.3	5.80	122.3	5.81	121.9	5.83	122.0
8	-	167.7	-	167.6	-	167.6	-	167.6	-	166.9	-	167.0
9	3.16	35.3	3.16	35.2	3.15	35.2	3.16	35.2	2.95	35.9	2.94	35.9
10	-	39.3	-	39.3	-	39.1	-	39.3	-	38.9	-	39.0
11α β	1.71 1.81	21.6	1.70 1.82	21.6	1.67 1.79	21.6	1.70 1.82	21.6	1.67 1.77	21.7	1.67 1.75	21.7
12α β	1.88 2.14	32.3	1.90 2.17	32.5	1.87 2.14	32.2	1.90 2.17	32.5	1.87 2.13	32.3	1.82 2.11	32.4
13	-	48.8	-	48.7	-	48.5	-	48.5	-	48.7	-	48.8
14	-	85.3	-	85.4	-	85.3	-	85.4	-	85.2	-	85.4
15α β	2.00 1.66	31.9	1.94 1.60	31.7	1.98 1.65	31.9	1.92 1.59	31.7	1.99 1.65	31.8	2.03 1.67	31.8
16α β	2.07 1.99	22.9	2.06 1.88	22.1	2.07 1.98	22.9	2.03 1.85	22.0	2.07 2.00	22.9	2.18 1.95	22.5
17	2.44	51.7	2.45	51.0	2.44	51.6	2.44	50.9	2.45	51.6	2.36	50.7
18	0.89	17.8	0.88	17.9	0.87	17.8	0.86	17.9	0.89	17.8	0.96	17.9
19	0.96	24.6	0.96	24.5	0.95	24.6	0.96	24.5	0.96	24.2	0.98	24.1
20	-	85.7	-	86.7	-	85.8	-	86.8	-	86.0	-	86.5
21	1.31	23.7	1.33	19.7	1.30	23.8	1.33	19.6	1.29	23.7	0.90	21.9
22	3.85	86.2	379	84.3	3.85	86.2	3.78	84.3	3.88	86.3	3.86	84.1
23	1.65	24.8	1.58	25.6	1.64	24.8	1.59	25.6	1.65	24.8	1.53	25.1
24	1.78 1.54	42.3	1.79 1.51	42.4	1.78 1.54	42.2	1.78 1.50	42.4	1.75 1.55	42.2	1.85 1.52	42.3
25	-	71.2	-	71.2	-	71.2	-	71.2	-	71.2	-	71.3
26	1.20	29.1	1.21	29.1	1.20	29.1	1.20	29.1	1.21	29.1	1.21	29.2
27	1.21	29.6	1.22	29.6	1.21	29.6	1.21	29.6	1.22	29.6	1.22	29.6
28	-	-	-	-	-	-	-	-	-	109.5	-	109.6
29	-	105.3	-	102.8	-	105.0	-	102.6	-	105.7	-	108.5
R^1^ 1	-	-	-	-	-	-	-	-	1.47	29.0	1.47	28.9
R^2^ 1	-	-	-	-	-	-	-	-	1.32	26.8	1.32	26.7
R^3^ 1 2 3 4 5 6	5.73	-	- 7.00 - - 6.79 6.89	133.4 111.1 149.1 148.6 116.0 120.4	5.75	-	- 7.36 6.99 - 6.99 7.36	134.3 128.8 115.8 160.7 115.8 128.8	5.80	-	1.53	30.0
R^4^ 1 2 3 4 5 6	- 7.03 - - 6.78 6.93	131.7 111.6 148.6 n.d. 115.9 121.5	5.92	-	- 7.38 6.98 - 6.98 7.38	132.7 129.4 115.7 160.8 115.7 129.4	5.94	-	- 7.47 7.36 7.36 7.36 7.47	140.4 128.0 129.3 130.1 129.3 128.0	- 7.47 7.28 7.22 7.28 7.47	148.0 125.9 128.8 1283 128.8 125.9

As published before [9], the 20,22-diol moiety of 20E is more reactive than the 2,3-diol, probably due to the free rotation of the 20,22-bond of 20E that allows the 20,22-dioxolane ring to form with less strain. This allowed us to selectively obtain the 20,22-mono-dioxolane derivatives **2**–**14**, or, depending on the amount of reagent and the reaction time, the 2,3;20,22-bis-homo-dioxolanes **17** and **21**–**25**. By utilizing the 20,22-monodioxolane ecdysteroids, another aldehyde or ketone could be coupled to position 2,3, resulting in several bis-hetero-dioxolane derivatives **26**–**33**. For this, however, gradually decreasing reactivity with the increase of the size of the reagent was a limiting factor: larger aldehydes or ketones (mainly those containing a substituted aromatic ring) could not be coupled at the 2,3-position. The 2,3-monodioxolane derivatives also appeared to be present as minor side-products of the reactions, and as a consequence of their low amount, only one such compound (compound **15**) was isolated and studied. To selectively obtain this kind of a compound (**16)** in a more reasonable yield, another, three-step approach was successfully applied: after protecting the 20,22-diol with phenylboronic acid, the 2,3-acetonide could be prepared, and removal of the 20,22 protecting group afforded the desired 2,3-monoacetonide in a one-pot procedure.

**Figure 2:**
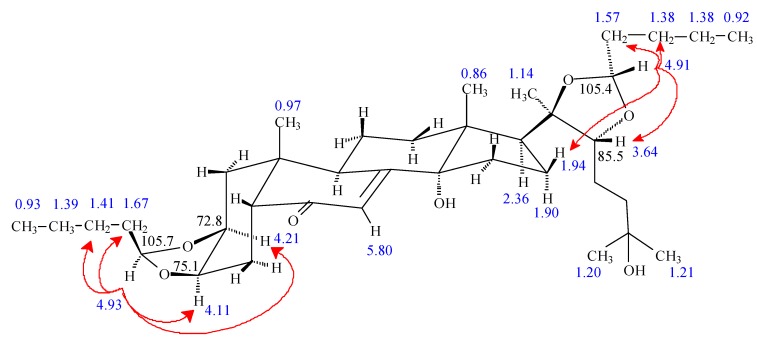
Stereostructure of **22**. Red arrows indicate the detected ROESY steric proximities, the blue numbers give the characteristic ^1^H, and the black numbers the ^13^C chemical shifts.

In the case of the reactions with aldehydes or asymmetric ketones, the new C-28 and C-29 central atoms of the dioxolane rings are stereogenic centers and thus two possible diastereomers can be formed at both diols. Their configuration was elucidated by two-dimensional ROESY or selective one-dimensional ROESY experiments, e.g., in the doubly substituted dioxolane derivative **22** (R^1^ = R^4^ = *n*-Bu, R^2^ = R^3^ = H) the unambiguous differentiation of the ^1^H and ^13^C signals of the two *n*-butyl groups was achieved in the following way (see Figure 2). Assignment of the H-C(28) atoms (*δ* = 4.93/105.9 ppm) was supported by the H-2/C-28 and H-3/C-28 HMBC correlations, and that of H-C(29) (*δ* = 4.91/105.6 ppm) by the H-22/C-29 cross peak, respectively. The selective ROESY experiment irradiating at 4.93 ppm showed contacts with the H_α_-2 and H_α_-3 atoms proving the α position of the R^2^ = H atom. The ROESY response obtained irradiating H = R^3^ signal (*δ* = 4.91) on H-22 (*δ* = 3.64 ppm) revealed their *cis* arrangement and the *R* configuration around C-29. The unambiguous assignments of the signals of the two *n*-butyl groups R^1^ and R^4^ were achieved by selective TOCSY experiments (irradiation at *δ* = 4.93 and 4.91, respectively).

In case of the C-28-epimers, typically an approximately 1:1 yield was obtained, and a good separation was achieved by simple chromatographic methods (see below). On the other hand, possibly due to steric reasons, the longer chain of the reagent was highly selective in taking the α-position in the 20,22-dioxolane moiety. This selectivity was, however, decreased in cases when larger moieties, such as substituted aromatic rings were present in the reagent, resulting in the appearance of the other epimers as well. These epimer pairs (compounds **11**-**12** and **13**-**14**) required high-performance liquid chromatography (HPLC) for their successful separation. Compound **10** was isolated by HPLC as a minor product from the preparation of **9**; this compound, considering the vicinal coupling constant of the olefinic hydrogen atoms (*J* = 11.8 Hz) contains a *Z* double bond, and most likely originated from an impurity in the *trans*-cinnamic aldehyde reagent used. Compounds **18** and **19** were the only cases where one of the dioxolane rings was formed with the elimination of ethanol instead of water, losing an ethyl group from the reagent methyl ethyl ketone.

### 2.2. Anti-Proliferative Effect of Ecdysteroid Derivatives on PAR and MDR Mouse Lymphoma Cells

The anti-proliferative activity of the derivatives was determined after 72 h of incubation of each of the cell lines with serial dilutions of the compounds. Inhibitory concentrations (IC_50_) were calculated and are presented in Table 4. As seen from the table, several compounds exert much lower anti-proliferative activity on the MDR cell line as compared to the parental one, while other compounds show similar activities on both cell lines.

### 2.3. Inhibition of the ABCB1 Pump of MDR Mouse Lymphoma Cells (Rhodamine 123 Accumulation Assay)

Accumulation of rhodamine 123 by MDR mouse lymphoma cells was evaluated by flow cytometry in the presence of the newly described compounds in order to study their capacity to inhibit the ABCB1 pump and therefore prevent the efflux of the dye, which was consequentially retained inside the MDR cell. Parental mouse lymphoma cells were used as control for dye retention inside the cell while MDR cells alone do not retain rhodamine 123 at the concentration employed. The efflux pump inhibitor (EPI) verapamil was used as positive control. All the compounds were dissolved in DMSO, which was also evaluated for any effect on the retention of the fluorochrome. DMSO concentration in the assay was 0.2%. For each compound, the fluorescence activity ratio (FAR), which measures the amount of rhodamine 123 accumulated by the cell in presence of the compound was calculated as follows:

FAR = (FL_MDRtreated_/FL_MDRuntreated_)/(FLPARtreated/FL_PARuntreated_) (1)

where FL is the mean of the fluorescence. The obtained results are shown by Table 4.

As seen from the table, the compounds showed marked differences according to their capacity to inhibit the efflux of rhodamine 123 in this bioassay: from the practically inactive (compounds **5**, **7**, **9**, **10**, **12** and **20**) to the very strong (compounds **6**, **8**, **14** and **25**), various activities were observed. Most interestingly, these results did not always conform to those obtained from the combination studies, for example, no significant differences can be observed between the combination indices of compounds **20** and **25**, and compound **3**, very weak in this assay, was able to act in a rather significant synergism with doxorubicin (see below). These observation seem to support our initial theory, that these compounds are not or not exclusively acting as EPIs, but other mechanisms may also be involved in their activity [7].

**Table 4:** IC_50_ values of the ecdysteroid derivatives and fluorescence activity ratio (FAR) values determined in presence of 2 and 20 μM of compound. IC_50_—inhibitory concentration (concentration of compound that inhibits 50% of cell growth); IC_50_ values are presented as the average of 3 independent experiments ± the standard error of the mean (SEM); *—the compound showed cytotoxicity at this concentration and it was not possible to calculate the FAR value; FAR values of the positive control verapamil (20.4 μM) and the negative control DMSO (0.2%) were 5.73 and 0.72, respectively.

Compound	IC_50_ (µM)		FAR	
	PAR	MDR	2 µM	20 µM
20E	>150	>150	1.70	1.76 [7]
**3**	40.6 ± 3.6	>150	1.02	4.04
**4**	21.4 ± 1.4	57.8 ± 5.7	2.40	40.76
**5**	77.0 ± 1.5	92.7 ± 2.0	1.42	1.40
**6**	18.5 ± 1.8	35.7 ± 0.5	1.53	98.74
**7**	50.0 ± 3.4	>150	1.04	1.69
**8**	34.1 ± 3.3	38.8 ± 0.4	1.21	94.56
**9**	36.7 ± 0.8	89.4 ± 14.4	0.77	1.01
**10**	45.0 ± 9.6	>150	0.76	0.91
**11**	92.3 ± 23.1	>150	0.87	7.85
**12**	~98.9	>150	0.87	0.87
**13**	31.6 ± 5.3	43.2 ± 2.7	1.11	*
**14**	41.0 ± 6.8	59.5 ± 5.8	1.00	109.40
**15**	98.0 ± 1.1	87.1 ± 9.3	43.43	14.23
**16**	75.13 ± 0.7	85.8 ± 10.6	0.79	3.68
**17**	51.2 ± 1.5	56.0 ± 6.4	29.96	45.35
**18**	99.6 ± 5.2	>150	6.47	53.49
**19**	52.6 ± 1.5	87.8 ± 10.9	10.00	55.81
**20**	68.9 ± 2.1	>150	0.95	1.04
**21**	19.5 ± 2.6	30.6 ± 1.4	43.81	*
**22**	19.9 ± 0.03	25.5 ± 3.4	114.64	*
**23**	52.6 ± 12.9	49.7 ± 3.9	14.71	*
**24**	20.3 ± 0.8	22.4 ± 0.8	51.97	*
**25**	30.2 ± 1.2	38.3 ± 1.1	1.08	82.68
**26**	>150	>150	3.88	14.07
**27**	>150	>150	1.41	17.67
**28**	75.2 ± 12.1	64.4 ± 13.7	1.68	60.67
**29**	77.5 ± 20.7	75.9 ± 3.1	2.21	68.46
**30**	72.2 ± 9.8	66.8 ± 4.7	51.67	75.17
**31**	42.0 ± 18.9	42.7 ± 2.6	10.98	67.78
**32**	41.6 ± 6.7	46.5 ± 7.0	61.67	*
**33**	62.6 ± 16.8	64.7 ± 7.3	3.47	63.22

### 2.4. Combination Studies: Effect of Ecdysteroid Derivatives on the Activity of Doxorubicin on MDR Mouse Lymphoma Cells

**Figure 3:**
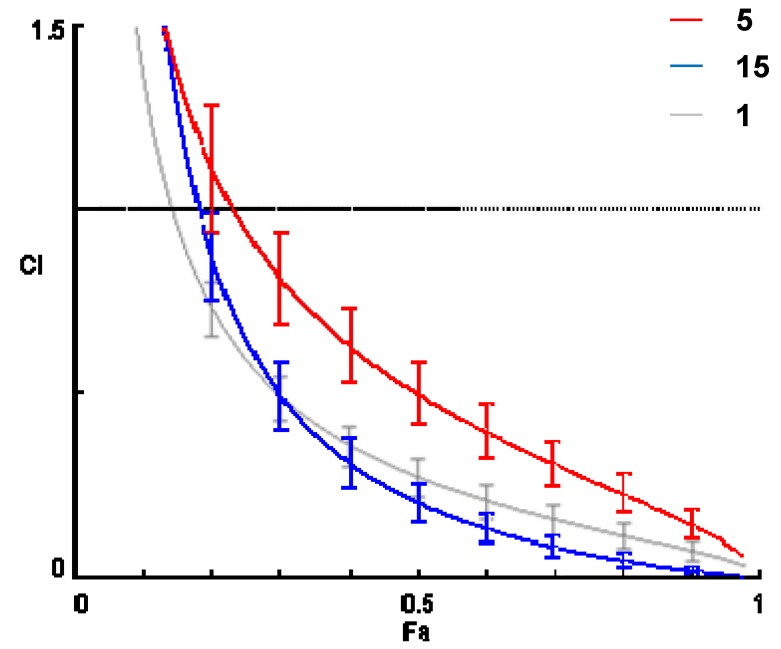
Fraction affected (Fa) *vs.* combination index (CI) value plot for compounds **5** and **15**, in comparison with the original lead compound **1**. Error bars represent 95% confidence intervals by means of serial deletion analysis performed with the CompuSyn software. The 2,3-mono-dioxolane derivative **15** represents significantly stronger synergism with doxorubicin than the corresponding 20,22-dioxolane derivative **5** at practically all activity levels, and above Fa = 0.7 (which, in case of cancer, matters the most [10]) it is also stronger than compound **1**.

Effect of the newly synthesized derivatives was evaluated on checkerboard 96-well plates with different concentrations of doxorubicin and compound after 48 h of incubation of the cells, similarly to our previous approach [7]. Combination indices for the different constant ratios of compound *vs.* doxorubicin were determined by using the CompuSyn software to plot four to five data points to each ratio. CI values were calculated by means of the median-effect equation [10], where CI < 1, CI = 1, and CI > 1 represent synergism, additive effect (*i.e.*, no interaction), and antagonism, respectively. The CI values are presented on Table 5. Combination index plots (or Fa-CI plots, where Fa is the fraction affected) were also generated for each compound using serial deletion analysis in order to determine variability of the data [10]. An example of Fa-CI plot is given by Figure 3 for compounds **1**, **5** and **15**.

**Table 5:** Combination index (CI) values at different drug ratios (compound *vs.* doxorubicin, respectively) at 50, 75 and 90% of growth inhibition (ED_50_, ED_75_ and ED_90_, respectively); CI_avg_—weighted average CI value; CI_avg_ = (CI_50_ + 2CI_75_ + 3CI_90_)/6. CI < 1, CI = 1, and CI > 1 represent synergism, additivity, and antagonism, respectively. Dm, m, and r represent antilog of the *x*-intercept, slope, and linear correlation coefficient of the median-effect plot, respectively.

Compound	Drug Ratio	CI Values at			Dm	m	r	CI_avg_
		ED_50_	ED_75_	ED_90_				
20E [7]	20.4:1	2.00	2.02	2.04	35.52	2.855	0.997	2.03
	40.8:1	1.86	1.97	2.10	54.05	2.487	0.978	2.02
	81.5:1	1.80	1.93	2.08	76.90	2.376	0.978	1.99
**1** [7]	20.4:1	0.28	0.14	0.07	11.68	3.246	0.964	0.13
	40.8:1	0.26	0.14	0.08	14.73	3.167	0.996	0.13
	81.5:1	0.39	0.22	0.12	26.60	3.859	0.970	0.20
**2** [7]	20.4:1	0.84	0.54	0.35	20.51	1.933	0.955	0.49
	40.8:1	1.22	0.86	0.61	48.83	1.766	0.947	0.79
	81.5:1	1.11	0.77	0.54	64.94	1.920	0.916	0.71
**3**	20.4:1	0.87	0.43	0.22	35.28	2.44	0.985	0.40
	40.8:1	0.76	0.31	0.13	45.44	3.54	0.962	0.29
	81.6:1	0.91	0.31	0.11	71.98	5.78	0.989	0.31
**4**	20.4:1	0.61	0.56	0.80	15.50	1.69	0.907	0.69
	40.8:1	0.56	0.58	0.78	18.06	1.95	0.870	0.68
	81.6:1	1.08	0.91	0.88	40.46	5.02	0.921	0.92
**5**	20.4:1	0.64	0.43	0.29	23.22	1.56	0.986	0.39
	40.8:1	0.50	0.26	0.14	29.50	2.25	0.959	0.24
	81.6:1	0.61	0.44	0.33	52.58	1.32	0.989	0.41
**6**	20.4:1	0.57	0.52	0.50	6.38	1.15	0.992	0.52
	40.8:1	0.99	0.59	0.36	13.92	2.38	0.981	0.54
	81.6:1	0.70	0.44	0.28	11.21	2.34	0.936	0.40
**7**	20.4:1	0.80	0.86	1.20	31.47	1.04	0.945	1.02
	40.8:1	0.84	0.81	0.91	40.24	1.36	0.967	0.87
	81.6:1	1.16	0.96	0.86	62.14	1.96	0.996	0.94
**8**	20.4:1	0.63	0.47	0.61	10.85	2.04	0.820	0.57
	40.8:1	0.84	0.64	0.69	20.03	3.68	0.990	0.70
	81.6:1	0.76	0.68	0.74	22.38	3.99	0.949	0.72
**9**	20.4:1	1.32	0.92	1.17	25.01	1.46	0.988	1.11
	40.8:1	0.89	0.66	0.76	25.78	2.04	0.968	0.75
	81.6:1	0.82	0.68	0.74	32.71	2.66	0.950	0.73
**10**	20.4:1	1.12	0.84	0.91	26.31	1.84	0.980	0.92
	40.8:1	0.92	0.81	0.94	31.74	2.01	0.991	0.89
	81.6:1	1.24	0.98	0.92	55.74	4.10	0.988	0.99
**11**	20.4:1	0.95	0.53	0.34	23.61	2.03	0.945	0.51
	40.8:1	1.04	0.71	0.56	44.10	1.76	0.990	0.69
	81.6:1	0.89	0.65	0.55	59.01	1.95	0.997	0.64
**12**	20.4:1	1.13	0.80	0.57	19.20	1.09	0.982	0.74
	40.8:1	0.86	0.48	0.27	26.10	1.44	0.958	0.44
	81.6:1	1.32	0.53	0.21	66.47	2.79	0.997	0.50
**13**	20.4:1	0.66	0.61	0.70	12.53	1.75	0.954	0.66
	40.8:1	0.87	0.74	0.73	21.71	2.79	0.961	0.76
	81.6:1	0.85	0.93	1.12	25.07	2.02	0.898	1.01
**14**	20.4:1	0.20	0.25	0.33	6.21	1.70	0.946	0.28
	40.8:1	0.26	0.30	0.35	10.74	2.43	0.992	0.32
	81.6:1	0.28	0.34	0.42	13.24	2.33	0.979	0.37
**15**	20.4:1	0.33	0.12	0.05	16.86	2.25	0.971	0.12
	40.8:1	0.27	0.10	0.04	24.03	2.61	1.000	0.10
	81.6:1	0.20	0.06	0.02	26.98	4.12	0.969	0.07
**16**	20.4:1	0.64	0.53	0.44	13.16	1.31	0.993	0.50
	40.8:1	0.67	0.35	0.19	23.63	3.09	0.989	0.33
	81.6:1	0.34	0.24	0.17	18.98	1.92	0.967	0.22
**17**	20.4:1	0.31	0.25	0.24	5.14	3.21	0.999	0.25
	40.8:1	0.37	0.35	0.36	7.80	2.96	0.996	0.36
	81.6:1	0.54	0.56	0.60	12.84	2.85	0.987	0.58
**18**	20.4:1	0.23	0.16	0.15	6.32	2.66	0.956	0.16
	40.8:1	0.27	0.23	0.24	10.03	2.48	0.984	0.24
	81.6:1	0.40	0.37	0.39	17.83	2.61	0.998	0.39
**19**	20.4:1	0.31	0.22	0.17	6.48	3.67	0.978	0.21
	40.8:1	0.36	0.31	0.27	9.15	2.77	0.970	0.30
	81.6:1	0.52	0.46	0.42	14.59	2.81	0.977	0.45
**20**	20.4:1	0.92	0.59	0.41	19.96	2.41	0.968	0.55
	40.8:1	0.72	0.58	0.50	23.55	1.86	0.984	0.56
	81.6:1	1.06	0.65	0.42	46.17	4.64	0.970	0.60
**21**	20.4:1	0.44	0.32	0.27	6.72	4.53	1.000	0.31
	40.8:1	0.43	0.40	0.40	7.82	2.88	0.944	0.40
	81.6:1	0.58	0.61	0.67	11.75	2.46	0.963	0.63
**22**	20.4:1	0.39	0.38	0.39	6.55	2.41	0.979	0.39
	40.8:1	0.52	0.50	0.49	9.80	2.85	0.992	0.50
	81.6:1	0.71	0.65	0.60	14.25	3.64	0.980	0.63
**23**	20.4:1	0.22	0.29	0.42	6.84	1.54	0.998	0.34
	40.8:1	0.26	0.30	0.35	10.74	2.43	0.992	0.32
	81.6:1	0.28	0.34	0.42	13.24	2.33	0.979	0.37
**24**	20.4:1	0.70	0.69	0.74	5.87	1.98	0.998	0.71
	40.8:1	0.79	0.82	0.88	7.25	2.02	0.989	0.85
	81.6:1	0.56	0.88	1.42	5.30	1.17	0.942	1.10
**25**	20.4:1	0.54	0.40	0.31	4.22	2.18	0.947	0.38
	40.8:1	0.95	0.64	0.43	7.74	2.99	0.995	0.59
	81.6:1	1.01	0.72	0.52	8.37	2.62	0.985	0.67
**26**	20.4:1	0.31	0.18	0.13	6.14	4.16	0.962	0.18
	40.8:1	0.37	0.25	0.20	10.51	4.37	0.991	0.25
	81.6:1	0.56	0.42	0.36	20.13	4.72	0.992	0.41
**27**	20.4:1	0.34	0.18	0.15	5.39	3.20	0.956	0.19
	40.8:1	0.31	0.22	0.21	7.32	2.73	0.981	0.23
	81.6:1	0.38	0.32	0.33	12.04	2.85	0.969	0.33
**28**	20.4:1	0.21	0.12	0.07	6.01	3.34	0.956	0.11
	40.8:1	0.20	0.13	0.09	9.21	3.03	0.991	0.12
	81.6:1	0.20	0.14	0.10	12.73	3.14	0.973	0.13
**29**	20.4:1	0.20	0.13	0.10	6.07	3.31	0.958	0.13
	40.8:1	0.19	0.14	0.12	8.23	3.39	0.963	0.14
	81.6:1	0.22	0.21	0.22	12.36	2.46	0.981	0.22
**30**	20.4:1	0.23	0.08	0.06	4.71	2.20	0.984	0.09
	40.8:1	0.21	0.10	0.07	6.98	2.32	0.994	0.11
	81.6:1	0.21	0.14	0.13	9.92	1.86	0.995	0.15
**31**	20.4:1	0.12	0.10	0.12	2.86	1.83	0.963	0.11
	40.8:1	0.13	0.15	0.21	4.43	1.66	0.969	0.17
	81.6:1	0.22	0.23	0.29	8.97	2.26	0.998	0.26
**32**	20.4:1	0.18	0.12	0.12	4.40	2.66	0.999	0.13
	40.8:1	0.16	0.16	0.20	5.22	1.80	0.990	0.18
	81.6:1	0.23	0.24	0.29	9.18	2.15	0.971	0.26
**33**	20.4:1	0.15	0.13	0.14	4.12	1.85	0.999	0.14
	40.8:1	0.14	0.15	0.22	5.14	1.52	0.982	0.18
	81.6:1	0.19	0.27	0.44	9.18	1.36	0.992	0.34

As seen from Table 5, all compounds acted synergistically with doxorubicin and their behavior followed our previous observation, namely that in case of all ecdysteroids there seems to be an “ideal” compound *vs.* doxorubicin ratio where the strongest synergistic effect occurs. Based on the variability of the mono-, homo-di- and hetero-di-substituted compounds, as well as that of the coupled substituents at R^1^–R^4^, several novel structure-activity relationships (SARs) were observed. According to this, we followed our previous approach [7]—for each compound, the strongest activity by means of the weighted average CI values was primarily considered for comparison, regardless of the compound *vs.* doxorubicin ratio where this activity was found.

**Figure 4:**
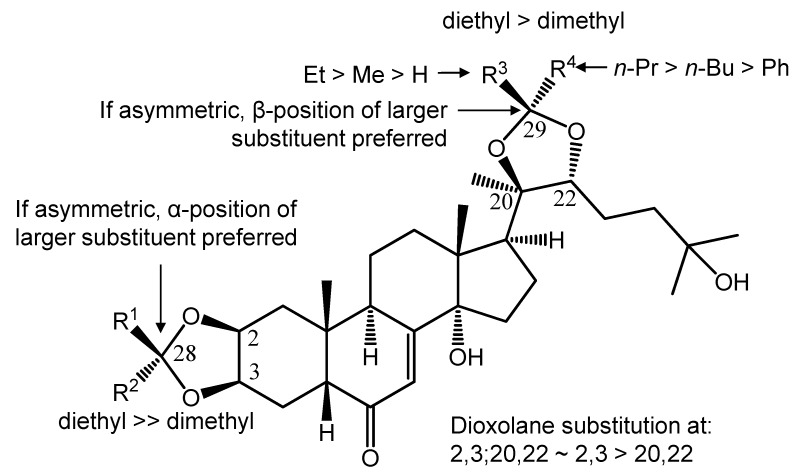
SAR summary for compounds **1**–**33**. “Greater than” symbols denote stronger synergistic activities, *i.e.*, lower weighted average CI values when applied together with doxorubicin.

First of all, as a surprising outcome of our experiments, the 2,3-dioxolane moiety is far more important for a strong activity, than the one at positions 20,22. In fact, compound **15**, monosubstituted at position 2,3, was the only ecdysteroid derivative in the present investigation that was able to exert a stronger activity at its best ratio than our original lead, the diacetonide compound **1** (Figure 3). A very interesting SAR was revealed by comparing the activity of the C-28 and C-29 epimer pairs: at C-28, the larger substituent needs to take the α-position (**24** *vs.* **25**), while at C-29 the β-position for a stronger activity (cf. **11** *vs.* **12** and **13** *vs.* **14**). As concerns the 20,22-monodioxolanes, increasing the length of the side chains coupled to C-29 lead to a significant increase in the synergistic activity with doxorubicin till the length of three carbon atoms (compound **3**), however a longer alkyl substituent (compound **4**) appeared to be less preferable. Introducing larger aromatic groups did not lead to a breakthrough, although further substituents on the aromatic ring (compounds **11**, **13**) were able to increase activity as compared to the case when a non-substituted phenyl group was present (compound **7**). Addition of a β-methyl group to C-29 could, however, significantly improve the activity as compared to that of the 29α-phenyl substituted derivative (cf. **8** *vs.* **7**, respectively). The observed structure-activity relationships are summarized in Figure 4.

## 3. Experimental

### 3.1. General Information

The starting material 20E (90%, originated from the roots of *Cyanotis arachnoidea*) was purchased from Shaanxi KingSci Biotechnology Co., Ltd. (Shanghai, China), and further purified by crystallization from ethyl acetate–methanol (2:1, *v/v*), so that purity of 20E utilized for the semi-syntheses was 97.8%, by means of HPLC-DAD, maximum absorbance within the range of 220–400 nm. Mono- and disubstituted ecdysteroid dioxolanes were synthesized as published before [9]. Briefly, the starting compound was reacted with the aldehyde or ketone to be coupled to positions 20,22 and/or 2,3 in the presence of phosphomolybdic acid (Lach-Ner, Neratovice, Czech Republic) at room temperature for 5–60 min depending on the target compound. The reaction was terminated by neutralizing the pH with a 5% aqueous solution of NaHCO_3_ (Merck, Munich, Germany), methanol was evaporated until only water was present, and the product(s) were extracted with methylene chloride. Column chromatography (CC), rotational planar chromatography (RPC) and/or crystallization was used for purification, as detailed below. Solvent system compositions are given in v/v%. For RPC, a Chromatotron device (Harrison Research, Palo Alto, CA, USA) was used. The separation was monitored with thin layer chromatography (TLC) on silica gel 60 F_254_ (0.25 μm, Merck). HPLC purification of compounds **9**–**14** was performed on a gradient system of two Jasco PU2080 pumps connected to a Jasco MD-2010 Plus photodiode-array detector, on a Zorbax XDB-C8 column (5 µm, 9.6 × 250 mm) at a flow rate of 3 mL/min. Mass spectra were recorded on an API 2000 triple quadrupole tandem mass spectrometer (AB SCIEX, Foster City, CA, USA) in positive mode with atmospheric pressure chemical ionization (APCI) ion source except for compound **29** which was measured with electron-spray ionization (ESI). ^1^H- (500.1 MHz) and ^13^C- (125.6 MHz) NMR spectra were recorded at room temperature on an Avance 500 spectrometer (Bruker, Billerica, MA, USA). For the examples of compounds **3**, **5**, **7**, **8**, **15**, **21**, **23**–**25**, **28** and **29**, structure elucidation of ecdysteroid dioxolanes by comprehensive one- and two-dimensional NMR methods has recently been discussed in detail elsewhere, including experimental details for the aforementioned compounds [9]. Regarding the new compounds, amounts of approximately 1–10 mg were dissolved in 0.1 mL of methanol-*d*_4_ and transferred to a 2.5 mm Bruker MATCH NMR sample tube. Chemical shifts are given on the *δ*-scale and are referenced to the solvent (MeOH-d_4_: *δ_C_* = 49.1 and *δ_H_* = 3.31 ppm). Pulse programs of all experiments (^1^H, ^13^C, DEPTQ, DEPT-135, sel-TOCSY, sel-ROE, sel-NOE, gradient-selected (gs) ^1^H,^1^H-COSY, edited gs-HSQC, gs-HMBC, ROESY) were taken from the Bruker software library. Most ^1^H assignments were accomplished using general knowledge of chemical shift dispersion with the aid of the proton-proton coupling pattern (^1^H-NMR spectra).

### 3.2. Semi-Synthesis and Purification *of Monosubstituted* Ecdysteroid Dioxolane Derivatives **2**–**16**

20E was dissolved in methanol (10 mL, Merck) to a final concentration of 100 mM or 25 mM in case of compounds **9**, **10**, **13**, **14**, and the corresponding reagent (**3**: butyraldehyde, **4**: valeraldehyde, **5**: 3-pentanone, **6**: methyl isobutyl ketone, 10 equivalents each; **7**: benzaldehyde, 5 g; **8**: acetophenone, 6 g; **9**, **10**: cinnamaldehyde, **11**, **12**: vanillin, **13**, **14**: 4-benzyloxybenzaldehyde, 10 equivalents each; **15**: 3-pentanone, *100 equivalents*; compound **15** was obtained as a side-product from the synthesis of **25**, see below) was added to the solution. Phosphomolybdic acid (1.00 g) was added (except in the case of the synthesis of **9** and **10**, when 0.50 g were added) and the mixture was stirred at room temperature for 10 min (except for **7**: 5 min, **8**: 60 min, **15**: 30 min). In the case of compound **16**, 20E was dissolved in methanol (10 mL) to a final concentration of 100 mM, and after adding phenylboronic acid (1 equivalent), the mixture was stirred for 30 min. Acetone (500 equivalents) and phoshomolybdic acid (0.5 g) were added to the mixture, and after 1 h stirring a solution of NaOH and H_2_O_2_ was added in order to remove the phenylboronate group. Then, the reaction was worked up as described above. Compounds **3**, **4**, **7**, **8**, a mixture of **9**-**10**, and compounds **15** and **16** were obtained from RPC on silica gel with appropriate solvent systems of ethyl acetate-ethanol-water (**3**, **4**) or cyclohexane-ethyl acetate (**7**, **8**, **9**-**10**, **15**, **16**). The purification of compounds **11**-**12** and **13**-**14** started with CC by using solvent systems of ethyl acetate-ethanol-water. Isomer pairs **9**-**10**, **11**-**12** and **13**-**14** were isolated by RP-HPLC (**9**, **10**: 75% CH_3_OH aq., 3 mL/min; **11**, **12**: 70% CH_3_OH aq., 3 mL/min; **13**, **14**: 80% CH_3_OH aq., 3 mL/min). Compounds **2**, **5** and **6** were recrystallized from acetonitrile without chromatographic purification. The yields were: **2** (236.6 mg, 45.43%), **3** (116.2 mg, 21.7%), **4** (142.8 mg, 26.0%), **5** (183.5 mg, 33.4%), **6** (71.9 mg, 25.2%), **7** (292.5 mg, 51.4%), **8** (196.8 mg, 33.8%), **9** (27.0 mg, 18.5%), **10** (13.9 mg, 9.4%), **11** (156.3 mg, 25.4%), **12** (67.0 mg, 10.9%), **13** (67.3 mg, 39.9%), **14** (33.7 mg, 20.0%), **15** (27.4 mg, 5.0%), **16** (13.3 mg, 10.2%).

### 3.3. Semi-Synthesis and Purification of Disubstituted Ecdysteroid Derivatives **17**–**25** in One-Step

20E (**17**–**20**: 200 mg; **21**–**25**: 480 mg) was dissolved in methyl-ethyl ketone (20 mL, compounds **17**–**20**) or methanol (10 mL) and the reagent was added to the solution (**21**: butyraldehyde, 100 equivalents, **22**: valeraldehyde, 100 equivalents, **23**: 3-pentanone, 100 equivalents, **24**, **25**: benzaldehyde, 5 g). Phosphomolybdic acid was added (**17**–**20**: 20 mg; **21**–**25**: 0.50 g), and the mixture was stirred at room temperature for 5 (**17**–**20**, **24**–**25**) or 30 (**21**–**23**) min. The reactions were worked up as described above, and the products were isolated by RPC using the appropriate *n*-hexane-acetone (**17**–**20**) or cyclohexane-ethyl acetate-ethanol (**21**–**25**) solvent systems. As a side-product of the reaction of 20E with methyl-ethyl ketone, **20** was obtained as a 20,22-monodioxolane derivative. The yields were: **17** (15.5 mg, 6.3%), **18** (4.9 mg, 2.1%), **19** (8.4 mg, 3.6%), **20** (4.46 mg, 2.0%) **21** (242.4 mg, 41.2%), **22** (134.5 mg, 21.8%), **23** (42.3 mg, 6.9%), **24** (36.1 mg, 5.5%), **25** (43.8 mg, 6.7%). 

### 3.4. Semi-Synthesis and Purification of Disubstituted Ecdysteroid Derivatives **26**–**33** in Two-Steps

Previously obtained 20,22-monosubstituted compounds (**2**, 20.7 mg; **3**, 40.0 mg; **5**, 40.7 mg; **6**, 50.0 mg; **7**, 57.0 mg; **8**, 87.3 mg; **2**, 104.0 mg) were dissolved in methyl ethyl ketone (2 mL, **26** and **27**) or in methanol (5 mL) and the reagent (**28**–**32**: acetone, 500 equivalents; **33**: butyraldehyde, 500 equivalents) was added to the solution. Phosphomolybdic acid (**26**, **27**: 20 mg; **28**–**32**: 0.5 g) was added to the solution, and the mixture was stirred at room temperature for 5 (**26**, **27**) or 60 (**28**–**33**) min. The reactions were terminated and the products were purified as described above for the disubstituted derivatives. The yields were: 26 (5.1 mg, 23.1%), 27 (5.1 mg, 23.1%), **28** (10.9 mg, 25.4%), **29** (15.8 mg, 36.2%), **30** (15.5 mg, 28.9%), **31** (24.8 mg, 40.6%), **32** (38.7 mg, 41.5%), **33** (53.0 mg, 46.2%).

### 3.5. Further Experimental Data for the New Compounds

*29α-Butyl-20,22-O-methylidene-20-hydroxyecdysone* (**4**): white needle-like crystals; mp 197–199 °C; for ^1^H- and ^13^C-NMR data, see Table 1; APCI-MS, *m/z* (I_rel_, %): 549 [M+H]^+^, 531 [M+H-H_2_O]^+^, 445, 427, 409.

*29α-I-butyl-29β-methyl-20,22-O-methylidene-20-hydroxyecdysone* (**6**): white needle-like crystals; mp 198–199 °C; for ^1^H- and ^13^C-NMR data, see Table 1; APCI-MS, *m/z* (I_rel_, %): 563 [M+H]^+^, 545 [M+H-H_2_O]^+^, 445, 427, 409.

*29α-E-ethenylbenzyl-20,22-O-methylidene-20-hydroxyecdysone* (**9**): white solid; mp. 161–163 °C; for ^1^H- and ^13^C-NMR data, see Table 1, in addition to this, the vicinal coupling constant of the olefinic hydrogen atoms (*J* = 16.0 Hz) proved the *E* configuration of the double bond; APCI-MS, *m/z* (I_rel_, %): 595 [M+H]^+^, 577 [M+H-H_2_O]^+^, 445, 427, 409.

*29α-Z-ethenylbenzyl-20,22-O-methylidene-20-hydroxyecdysone* (**10**): white solid; mp 138–140 °C; for ^1^H- and ^13^C-NMR data, see Table 1; APCI-MS, *m/z* (I_rel_, %): 595 [M+H]^+^, 577 [M+H-H_2_O]^+^, 445, 427, 409.

*29α-(3-Methoxy-4-hydroxyphenyl)-20,22-O-methylidene-20-hydroxyecdysone* (**11**): white solid; mp 163–165 °C; for ^1^H- and ^13^C-NMR data, see Table 3; APCI-MS, *m/z* (I_rel_, %): 615 [M+H]^+^, 597 [M+H-H_2_O]^+^, 445, 427, 409.

*29β-(3-Methoxy-4-hydroxyphenyl)-20,22-O-methylidene-20-hydroxyecdysone* (**12**): white solid; mp 157–159 °C; for ^1^H- and ^13^C-NMR data, see Table 3; APCI-MS, *m/z* (I_rel_, %): 615 [M+H]^+^, 597 [M+H-H_2_O]^+^, 445, 427, 409.

*29α-(4-Benzyloxyphenyl)-20,22-O-methylidene-20-hydroxyecdysone* (**13**): white solid; mp 144–146 °C; for ^1^H- and ^13^C-NMR data, see Table 3; APCI-MS, *m/z* (I_rel_, %): 675 [M+H]^+^, 445, 427, 409.

*29β-(4-Benzyloxyphenyl)-20,22-O-methylidene-20-hydroxyecdysone* (**14**): white solid; mp 139–141 °C; for ^1^H- and ^13^C-NMR data, see Table 3; APCI-MS, *m/*z (I_rel_, %): 675 [M+H]^+^, 445, 427, 409.

*20-Hydroxyecdysone 2,3-acetonide* (**16**): white solid; mp 124–126 °C; for ^1^H- and ^13^C-NMR data, see Table 2; APCI-MS, *m/z* (I_rel_, %): 553 [M+H+MeOH]^+^, 535, 503, 485, 467, 409.

*28α,29α-Diethyl-28β,29β-dimethyl-2,3;20,22-bis-O-methylidene-20-hydroxyecdysone* (**17**): white solid; mp 98–100 °C; for ^1^H- and ^13^C-NMR data, see Table 2; APCI-MS, *m/z* (I_rel_, %): 589 [M+H]^+^, 571 [M+H-H_2_O]^+^, 499, 481, 409.

*28α,29α-Dimethyl-28β-ethyl-2,3;20,22-bis-O-methylidene-20-hydroxyecdysone* (**18**): white solid; mp 99–101 °C; for ^1^H- and ^13^C-NMR data, see Table 2; APCI-MS, *m/z* (I_rel_, %): 561 [M+H]^+^, 543 [M+H-H_2_O]^+^, 499, 481, 409.

*28β,29β-Dimethyl-29α-ethyl-2,3;20,22-bis-O-methylidene-20-hydroxyecdysone* (**19**): white solid; mp 79–81 °C; for ^1^H- and ^13^C-NMR data, see Table 2; APCI-MS, *m/z* (I_rel_, %): 561 [M+H]^+^, 543 [M+H-H_2_O]^+^, 471, 453, 409.

*29α-Ethyl-29β-methyl-20,22-O-methylidene-20-hydroxyecdysone* (**20**): white solid; mp 140–142 °C; for ^1^H- and ^13^C-NMR data, see Table 2; APCI-MS, *m/z* (I_rel_, %): 535 [M+H]^+^, 517 [M+H-H_2_O]^+^, 445, 427, 409.

*28β,29α-Dibutyl-2,3;20,22-bis-O-methylidene-20-hydroxyecdysone* (**22**): transparent crystals; mp 186–187 °C; for ^1^H- and ^13^C-NMR data, see Table 1; APCI-MS, *m/z* (I_rel_, %): 617 [M+H]^+^, 599 [M+H-H_2_O]^+^, 513, 495, 409.

*28β,29,29-Trimethyl-2,3;20,22-bis-O-methylidene-20-hydroxyecdysone* (**26**): white solid; mp 100–102 °C; for ^1^H- and ^13^C-NMR data, see Table 2; APCI-MS, *m/z* (I_rel_, %): 547 [M+H]^+^, 517, 499, 467, 409.

*28α,29,29-Trimethyl-28β-ethyl-2,3;20,22-bis-O-methylidene-20-hydroxyecdysone* (**27**) white solid; mp 360–362 °C; for ^1^H- and ^13^C-NMR data, see Table 2; APCI-MS, *m/z* (I_rel_, %): 575 [M+H]^+^, 557 [M+H-H_2_O]^+^, 499, 481, 409.

*28,28,29β-Trimethyl-29α-i-buthyl-2,3;20,22-bis-O-methylidene-20-hydroxyecdysone* (**30**): transparent solid; mp 114–115 °C; for ^1^H- and ^13^C-NMR data, see Table 1; APCI-MS, *m/z* (I_rel_, %): 603 [M+H]^+^, 585 [M+H-H_2_O]^+^, 485, 467, 409.

*28,28-Dimethyl-29α-phenyl-2,3;20,22-bis-O-methylidene-20-hydroxyecdysone* (**31**): white solid; mp 114–117 °C; for ^1^H- and ^13^C-NMR data, see Table 3; APCI-MS, *m/z* (I_rel_, %): 641 [M+H+MeOH]^+^, 623, 517, 485, 467, 409.

*28,28,29β-Trimethyl-29α-phenyl-2,3;20,22-bis-O-methylidene-20-hydroxyecdysone* (**32**): white solid; mp 126–128 °C; for ^1^H- and ^13^C-NMR data, see Table 3; APCI-MS, *m/z* (I_rel_, %): 623 [M+H]^+^, 605 [M+H-H_2_O]^+^, 485, 467, 409.

*28β-Propyl-29,29-dimethyl-2,3;20,22-bis-O-methylidene-20-hydroxyecdysone* (**33**): transparent solid; mp 109–111 °C; for ^1^H- and ^13^C-NMR data, see Table 1; APCI-MS, *m/z* (I_rel_, %): 575 [M+H]^+^, 557 [M+H-H_2_O]^+^, 499, 481.

### 3.6. Preparation of the Compounds for the Bioassays

Each compound was dissolved in 99.5% DMSO (Sigma, Munich, Germany). In each protocol DMSO was always tested as solvent control and no activity was observed.

### 3.7. Cell Lines

Two mouse lymphoma cell lines were used in this work: a parental (PAR) cell line, L5178 mouse T-cell lymphoma cells (ECACC catalog no. 87111908, U.S. FDA, Silver Spring, MD, USA); and a multi-drug resistant (MDR) cell line derived from PAR by transfection with pHa MDR1/A retrovirus [11]. MDR cell line was selected by culturing the infected cells with 60 μg/L colchicine. Both cell lines were cultured in McCoy’s 5A medium supplemented with 10% heat inactivated horse serum, L-glutamine, and antibiotics (penicillin and streptomycin) at 37 °C and 5% CO_2_ atmosphere [12]. Medium, horse serum, and antibiotics were purchased from Difco (Detroit, MI, USA).

### 3.8. Anti-proliferative Assay

Anti-proliferative activities on PAR and MDR cell lines were performed as described before [7]. Briefly, 6 × 10^3^ cells/well were incubated with serial dilutions of each compound (*n* = 3) in McCoy’s 5 A medium for 72 h at 37 °C, 5% CO_2_. Then, MTT (Sigma) [13] was added to each well at a final concentration of 0.5 mg/mL per well) and after 4 h of incubation, 100 µL of SDS 10% (Sigma) in 0.01 M HCl was added to each well. Plates were further incubated overnight and optical density at 540 and 630 nm using an ELISA reader (Multiskan EX, Thermo Labsystem, Milford, MA, USA). Fifty percent inhibitory concentrations (IC_50_) were calculated using non-linear regression curve fitting of log(inhibitor) *vs.* response and variable slope with a least squares (ordinary) fit of GraphPad Prism 5 software (GraphPad Software, Sand Diego, CA, USA).

### 3.9. Inhibition of ABCB1 Pump of MDR Mouse Lymphoma Cells (Rhodamine 123 Accumulation Assay)

Inhibition of ABCB1 was evaluated using rhodamine 123, a fluorescent dye, which retention inside the cells was evaluated by flow cytometry (14). Briefly, 2 × 10^6^ cells/mL were treated with 2 and 20 μM of each compound. After 10 min incubation, rhodamine 123 (Sigma) was added to a final concentration of 5.2 μM and the samples were incubated at 37 °C in water bath for 20 min. Samples were centrifuged (2,000 rpm, 2 min) and washed twice with phosphate buffer saline (PBS, Sigma). The final samples were re-suspended in 0.5 mL PBS and its fluorescence measured with a Partec CyFlow flow cytometer (Partec, Münster, Germany). Verapamil (Sanofi-Synthelabo, Budapest, Hungary) at 20.4 μM was used as positive control.

### 3.10. Combination Assays

The combined activity of doxorubicin (Teva, Budapest, Hungary) and the ecdysteroids was determined using the checkerboard microplate method, as described before [7]. Briefly, 5 × 10^4^ cells/well were incubated with doxorubicin and the compound to be tested for 48 h at 37 °C under 5% CO_2_. Cell viability rate was determined through MTT staining, as described above. The interaction was evaluated using the CompuSyn software (CompuSyn Inc., Paramus, NJ, USA) at each constant ratio of compound *vs.* doxorubicin (M/M), and combination index (CI) values were obtained for 50%, 75%, and 90% of growth inhibition.

## 4. Conclusions

In the present study, we have prepared 32 semi-synthetic derivatives of 20-hydroxyecdysone, following our previously observed structure-activity relationships on the strong synergistic activity of ecdysteroid dioxolanes with doxorubicin on a murine MDR cancer cell line expressing the human ABCB1 transporter. By utilizing the different reactivity of the 2,3 and 20,22 vicinal diol moieties, various bis-homo- and bis-hetero-dioxolanes were synthesized, as well as several 20,22- and two 2,3-monodioxolane derivatives. In addition to these, two epimer pairs were also obtained. Twenty compounds are reported for the first time; their chemical structures were thoroughly investigated by comprehensive 1 and 2D-NMR methods, based on which complete signal assignments are provided.

The compounds showed mild to very strong synergistic effects with doxorubicin against the aforementioned MDR cancer cell line, and the diversity of the substituents allowed us to observe several new structure-activity relationships. Among these, the importance of the 2,3-dioxolane substitution and the observations concerning the role of stereochemistry at C-28 and C-29 are the most interesting results. Apparently, ecdysteroids can be engineered to become strong MDR modulators only by decreasing the polarity at the A-ring, while the polar side-chain can be kept, providing the possibility for designing such compounds with a reasonable water solubility and high drug-likeness.

Considering the high importance of the 2,3-dioxolane group in our compounds and the fact that exactly this part is the most sensitive to an acidic environment, *per os* application of these compounds requires an appropriate formulation; development of such delivery systems is currently in process, investigation on their activity against MDR cancer xenografts is going to be reported in the near future.
